# Multivariable Projections of Caries-Free Prevalence and the Associated Factors from 2019 to 2030 among Schoolchildren Aged 6, 12 and 16-Year-Old in Malaysia

**DOI:** 10.3390/children10071125

**Published:** 2023-06-28

**Authors:** Lokman Najihah, Wan Zakiyatussariroh Wan Husin, Jamaludin Marhazlinda

**Affiliations:** 1 Department of Community Oral Health and Clinical Prevention, Faculty of Dentistry, Universiti Malaya, Kuala Lumpur 50603, Malaysia; 2 Mathematical Science Studies, College of Computing, Informatics and Media, Universiti Teknologi MARA Cawangan Kelantan, Machang 18500, Malaysia

**Keywords:** caries free prevalence, schoolchildren, time-series, projection, gross domestic product, sugar consumption, water fluoridation

## Abstract

This study identified caries-free associated factors and conducted multivariable projections of the caries-free prevalence until 2030 among six-, 12-, and 16-year-old schoolchildren in Malaysia. It was a secondary data analysis of caries-free prevalence and potential associated factors obtained from the Health Information Management System (HIMS), Department of Statistics Malaysia (DOSM), and Food Balance Sheets (FBS). Multiple linear regression and regression with ARMA errors were employed to determine the associated factors and predict the caries-free prevalence from 2019 or 2020 until 2030 for the six-, 12-, and 16-year-old groups, respectively. Gross Domestic Product (GDP) and household income, sugar consumption, and water fluoridation were significantly associated with caries-free status, with the most impactful in all age groups being water fluoridation. With the projected values of the associated factors, the caries-free prevalence in schoolchildren of all age groups in Malaysia is predicted to increase in the next decade. Similar to the past decade, the prevalence trend will remain the highest among the 12-year-olds and the lowest among six-year-olds. Caries-free prevalence was predicted to increase by 9.10%, 15.52%, and 15.10% in the six-, 12-, and 16-year-old groups, respectively. The prevalence multiplied the highest at four times greater than in the past ten years among 16-year-olds, compared with less than 2% in the six- and 12-year-old groups. In conclusion, by factoring in economic factors, sugar consumption, water fluoridation, and age groups, the caries-free prevalence of schoolchildren in Malaysia is projected to increase at different rates in the next decade until 2030. Thus, strategic oral health plans to recognise effective promotion programmes and strengthen others for each age group are crucial.

## 1. Introduction

Dental caries affects 97% of the world’s population, with only 3% unaffected by this disease at any point during their lifetime [1]. Dental caries results from microbiologic shifts within the dental plaque, which cause the dissolution of the mineral structure on the hard dental tissues [2,3]. Children are most vulnerable to dental caries due to the fragility of their baby teeth and a lack of understanding of oral hygiene [4,5]. Affecting 60–90% of children worldwide, the impacts not only interfere with speech, self-confidence, and daily living activities but also affect essential nutrition intake, resulting in underweight children with abnormal cognitive development [6]. However, caries is mainly preventable despite being a very prevalent oral disease. Therefore, dentistry practise should be shifted to preventive-oriented strategies and directed towards a “caries-free future” [7].

A high incidence of dental caries was reported in middle-income countries with high sugar consumption [4]. As a developing country, Malaysia also reported higher than average consumption of sugar, with 19 teaspoons per day instead of the 6–9 teaspoons of sugar recommended by the United States Food and Drug Administration [8,9]. It was further discovered that young children in Malaysia were more likely to have caries when they consumed sugary foods and beverages more frequently than three times per day, above the World Health Organisation (WHO) limit [10,11]. According to Sheiham & James [12], one of the major reasons for ineffective control of caries is that most dental researchers and planners have concentrated on preventing caries at the individual level while ignoring the fundamental point that the severity of caries affects not only individuals but the population as well. Hence, a population-based initiative that needs to be prioritised is reducing the country’ sugar consumption per capita, which is the average individual intake of raw sugar in a specific nation [4,13].

At the same time, national wealth and economic growth are significant factors in determining health, and research consistently links higher healthcare spending to better health results. For example, a study found that a 1% increase in public health spending significantly reduced a 2.2% loss in standardised years of life [14]. Unfortunately, treatment and care for oral diseases are expensive [15]. Oral diseases are the fourth most costly to treat in most developed countries, involving 10% of public health expenditures. In developing countries, investments in oral health are minimal, and the cost of managing oral diseases alone will exceed all the available resources. While in emerging countries, almost all cases remained untreated [16,17]. According to the World Health Organisation [18], people from poor backgrounds are more vulnerable to oral diseases such as dental caries, periodontal disease, and oral cancer. Their economic situation affects their diet preference and oral health practises such as toothbrushing behaviour, reflecting oral health disparities between different social classes [19].

Simultaneously, fluoride has improved the prevalence of reported caries-free globally. The implementation of public water fluoridation disseminates the benefits to the entire community regardless of income, education level, age, or race, especially among those with poor oral care. A review of studies in 10 countries reported that fluoride has successfully decreased caries occurrence by 30–59% in primary teeth and 40–49% in permanent teeth among the population aged between three to 44 years old [20]. In addition, it helps reduce oral health inequalities between social classes [21].

In sum, a few important risk and protective factors for caries that also affect caries-free include sugar consumption, toothbrushing behaviour, economic factors, and fluoride exposure. However, due to the unavailability of periodic data for oral health behaviours, only sugar consumption, economic factors, and water fluoridation were considered in this study. This pioneering projection study focuses on enhancing the oral health of schoolchildren in Malaysia by producing essential data to achieve Fédération Dentaire Internationale (FDI) World Dental Vision objectives to provide optimal and comprehensive oral healthcare for all by 2030 [22]. Thus, this study has projected the caries-free prevalence, factoring in the associated factors among schoolchildren until 2030. The study of trends and projections of caries-free status among children is significantly valuable in caries research because caries progression is cumulative and early caries is an indicator of lifelong cavities. The information is crucial for policymaking, preventive and promotion programme planning, education, and research. This includes revising existing oral health programmes for schoolchildren and reorienting services towards prioritising preventive approaches that target in-need schoolchildren based on the evidence. More importantly, the forecast data on caries-free prevalence among schoolchildren will allow policymakers to estimate oral healthcare costs and prioritise more resources for vulnerable populations.

Additionally, one of the advantages of employing time-series analysis is its ability to produce more accurate predictions since it can account for correlated data series. As the observations that were measured repeatedly tend to be interrelated, typical inference methods are invalid due to the violation of the assumption of uncorrelated error, resulting in inaccurate estimations [23]. Therefore, this study employed a multivariable time series in projecting the caries-free prevalence from 2019 or 2020 until 2030 for six, 12-, and 16-year-old schoolchildren, factoring in the associated factors, which could provide a better and more accurate projection study.

## 2. Materials and Methods

### 2.1. Study Design and Participants, Data Source and Description

This study consists of a secondary data analysis of data obtained from the Health Information Management System (HIMS), the Department of Statistics Malaysia (DOSM), and the Food Balance Sheets (FBS), United Nations. HIMS is a database for healthcare delivery programmes where the system gathers all the regular reports related to healthcare services, including oral health, which were collected at the operational level (school and clinic). In addition, HIMS collects data on primary oral healthcare, specialist oral healthcare, and community oral healthcare for all age groups that receive oral healthcare delivery under the Ministry of Health (MOH). The data from the system was handled, analysed, and reported annually by the Information Documentation System (IDS) unit, MOH [24]. Meanwhile, DOSM is the primary government agency under the Ministry of Economic Affairs entrusted with collecting, analysing, interpreting, and disseminating the latest data and information regarding the country’s economic performance and social aspects [25]. Meanwhile, Food Balance Sheet (FBS) is an aggregated dataset that provides complete information about the food supply for 180 countries within a specific time frame, which includes all probable sources of supply/utilisation, food disappearance data, or a food consumption estimate of a certain food product within each country, created by the United Nations’ Food and Agriculture Organisation (FAO) in collaboration with the National Statistics Bureau [26].

The outcome of this study was mean caries-free prevalence (percentage of caries-free) among six-, 12-, and 16-year-old schoolchildren in Malaysia. Dental caries-free means the absence of any visible signs of dental caries in the mouth [27]. Children with a DMFT/dmft score of 0 (decayed, missing due to caries, and filled teeth) are considered caries-free. The prevalence of caries-free is calculated as the total number of schoolchildren with caries-free over the total number of schoolchildren attendances, multiplied by 100. The data on caries-free prevalence was extracted from the HIMS reports, consisting of 24 years of data points (1996–2019) among 12- and 16-year-olds, and 23 years of data points (1996–2018) among six-year-old schoolchildren. There were no rules of thumb for the minimum sample size required in a time-series analysis. The only theoretical limit available is having more data points than the number of parameters in the data [23]. The highest number of parameters in this study is very minimal. For example, in multiple linear regression among six-, 12-, and 16-year-old schoolchildren in Malaysia, the number of parameters is 5, consisting of an intercept and four regression coefficients. Hence, the allowed sample size to estimate the model can be as few as 5. Therefore, even with the short data series, this study’s number of data points is sufficient to perform a time-series analysis and is capable of producing a reasonable projection of cases as the number of observations is greater than the number of parameters.

Any missing value, extreme value, wrong data entry, or redundant data were checked and imputed using the linear interpolation method. The previous study showed that linear interpolation is an efficient imputation technique for time series data. Even though with 60% of missingness, the linear interpolation technique could produce minimal errors, with a mean absolute percentage error (MAPE) of 5.55% and a root mean square error (RMSE) of 1.06. [28]. In this study, some data points on household income and poverty incidence were unavailable in the 12 and 16 age groups (missingness = 58.3%) and the six age groups (missingness = 60.9%).

Furthermore, the factors associated with caries-free, which also refer to or are similar to the risk and protective factors for dental caries, including sugar consumption, economic factors, and water fluoridation coverage, with long periodical data available, were accounted for in the projection of the caries-free prevalence. Data on caries-free prevalence and water fluoridation coverage over the years in Malaysia was retrieved from the HIMS reports. Water fluoridation coverage is counted as the percentage of the population covered by the water fluoridation programme for a particular year. Finally, the socioeconomic indicators of Malaysia, such as gross domestic product (GDP), consumer price index (CPI), median household income, and relative poverty incidence, were extracted or downloaded from the DOSM website. GDP is an indicator that reflects a country’s economic performance and financial status. It measured the value of all finalised goods and services produced within the geographical boundaries of a country at a given period of time (usually one year) [29]. In Malaysia, there are three ways to calculate GDP, namely the production method (the total value added), the expenditure approach (the total of final expenditures), and the income approach (the total of the earnings received by the resident producer unit). Meanwhile, CPI is the index used to measure the change over time in the price paid by the consumer for a basket of consumer goods and services. Particularly, it is a scale for consumers’ inflation rate and purchasing power [29]. CPI is calculated based on the international standard and procedures known as the Laspeyres chain index method. This study extracted data regarding GDP per capita and CPI at a constant price from 1996 to 2019. Meanwhile, household income refers to the total incomes received by members of households that repeatedly occur within the reference period. In this study, the median household income among the Malaysian population was extracted instead of the mean income because it reflects the average Malaysian population’s earnings, while relative poverty incidence is counted as the percentage of households receiving 50% less than the average median income. Data on median household income per family and relative poverty incidence were obtained from a household survey conducted by the DOSM.

In the meantime, data regarding sugar consumption from 1996 to 2019 were retrieved from FBS provided by the Food and Agriculture Organisation of the United Nations (FAOSTAT) website. Nevertheless, as FBS only estimates the food available for the individual without considering the food wasted in each household, it might overestimate food consumption for the average resident as they did not include food losses during the estimation [30]. Despite inherent inaccuracies in estimating food consumption, the information on food supply patterns from FBS is crucial as an indicator of food consumption [9]. Other studies also extracted the sugar supply data at the population level to indicate sugar intake data for a specific age group, as reported by Masood, et al. [31]. The study was conducted across 73 countries and investigated the impact of national income and economic inequality in high- and low-income countries on the association between dental caries and sugar consumption among 12-year-old children. In this study, the category of sugar includes sugar cane, sugar beet, sugar (raw), and sweeteners (maple syrup, other fructose, syrup, glucose, lactose, non-alcoholic beverages, molasses, pure fructose, and pure maltose), which were measured in kcal/capita/day. The estimated per capita supply estimate for each food item (in terms of quantity and, using food conversion factors, in terms of calorie content, protein, and fat content) was calculated by dividing by the country’s population. A country’s total daily dietary energy supply (DES) per capita is then calculated by adding these per capita estimations of calorie content for individual food sources [32].

The Medical Ethics Committee, Faculty of Dentistry, the University of Malaya (DF CO2009/0035 (L), approved this research. It was a secondary data analysis of data obtained from the Health Information Management System (HIMS), the Department of Statistics Malaysia (DOSM), and the Food Balance Sheets (FBS). HIMS is a database for healthcare delivery programmes where the system gathers all the regular reports related to healthcare services, including oral health, which were collected at the operational level (school and clinic).

### 2.2. Data Analysis

In this study, we used the Pearson correlation test, Multiple Linear Regression (MLR), and MLR with ARMA error to project the caries-free prevalence among six-, 12-, and 16-year-old schoolchildren up to 2030. Using the Pearson correlation tests, only factors with a *p*-value less than 0.25 were included in the MLR. A cut-off point of 0.25 for further multivariable analysis is supported by a few articles [33,34,35]. As individual variables might significantly contribute when combined, the traditional cut-point of 0.05 for variable selection may result in removing essential variables from the model [34]. A value of +1 or −1 of Pearson’s correlation coefficient (r) indicates a perfect correlation between two variables. The absence of the correlation is indicated by 0 [36]. The significant associated factors were checked for multi-collinearity using the variance inflation factor (VIF) test. The VIF score of 1 and more than 10 points indicates the absence or presence of high multi-collinearity, which has caused an inaccurate estimation of the regression coefficient [37]. In the end, only associated factors with a *p*-value of correlation less than 0.25 and no possible presence of high multi-collinearity were included in the MLR analysis.

MLR model provides the linear relationship between the dependent variable, *y* with two or more predictor variables, x which can be explained as:

$$y_{t}=\beta_{0}+\beta_{1}x_{1}+\beta_{2}x_{2}+\ldots +\beta_{k}x_{k}+\varepsilon_{t}$$

where the coefficients, $\beta_{0},\;\beta_{1}$ …$\beta_{k}\;$ indicate the intercept and the slope of the line, respectively. The intercept, $\beta_{0}$ denotes the value of the *y* when x = 0. Meanwhile, the slope, $\beta_{1}$ …$\beta_{k}$ represents the changes of y by 1-unit changes of x. As for the error term, $\varepsilon_{t}$ it can be assumed to be the random error, which is present when there is a deviation from the straight line [23].

In regression analysis, it is very common for the errors to have a time series structure, violating the assumption of independent errors [38]. The presence of the serial correlation produces the wrong estimation of the coefficient and standard errors. The *p*-values associated with the coefficients may become too small, and some predictors become significant when they are not. Thus, the errors were adjusted with a time series structure using MLR with autoregressive moving average (ARMA) errors, which were assumed to follow an ARMA structure [23]. The equation of MLR with ARMA errors can be defined as:

$$y_{t}=\beta_{0}+\beta_{1}x_{1}+\beta_{2}x_{2}+\ldots +\beta_{k}x_{k}+\eta_{t}\;\text{where}\;\eta_{t}=\phi_{1}\eta_{t-1}+\varepsilon_{t}(\text{ARMA}\;\text{errors})$$

which is equivalent to regression model, where $\eta_{t}$ is an ARMA error.

$$y_{t}=\beta_{0}+\beta_{1}x_{1}+\beta_{2}x_{2}+\ldots +\beta_{k}x_{k}+\eta_{t}$$

In this study, the estimation and selection of the best regression model with ARMA errors was performed using the auto.arima command in R software version 4.0.4 (R Core Team, Vienna, Austria) via the xreg argument for the specified predictors.

## 3. Results

The present study examined caries-free prevalence data of 10,515,676 six-year-olds (from 1996 to 2018) and 11,127,663 12-year-olds and 7,820,688 16-year-old school children (from 1996 to 2019) attending Malaysian public schools, comprising around 70–80% of total children in Malaysia [39]. The school coverage for primary and secondary school children ranged from 81.75% (the lowest) to 97.3% (the highest) in primary school and 56.86% (the lowest) to 91.0% (the highest) in secondary school.

### 3.1. Selection of Potential Associated Factors of Caries-Free for MLR

All the factors demonstrated a significant correlation with caries-free prevalence, with a very good to perfect positive correlation between GDP, CPI, median household income, and water fluoridation coverage and caries-free prevalence for all the age groups. While relative poverty incidence and sugar consumption exhibited fair to moderate inverse correlations with caries-free prevalence. Only sugar consumption showed a non-significant correlation with caries-free in the 16-year-old group. The result is summarised in Table 1.

After removing the variable with the highest multi-collinearity, the process was repeated in the subsequent model until the remaining variables demonstrated no multi-collinearity with a VIF score of less than 10. The final model shows that household income, relative poverty, sugar consumption, and water fluoridation coverage demonstrated no multi-collinearity in the 6-year-old group, while GDP, relative poverty, sugar consumption, and water fluoridation coverage presented no multi-collinearity in the 12- and 16-year-old groups; hence, these variables were included in the MLR model for projecting the caries-free prevalence up to 2030.

### 3.2. Multiple Linear Regression of Caries-Free Prevalence and Associated Factors among Schoolchildren

MLR demonstrated that relative poverty incidence was not significantly associated with caries-free among six-year-olds (*p*-value = 0.828), 12-year-olds (*p*-value = 0.532), and 16-year-olds (*p*-value = 0.166), thus being removed from the projection of caries-free prevalence for all the age groups. However, other variables were significantly associated with caries-free prevalence (*p*-value < 0.05). The summary of the final model with the identified significant associated factors of caries-free for each group is in Table 2.

Upon checking the assumptions, the errors were found to be serially correlated in the 12-year-old age group (Supplementary Materials, Table S1). Thus, errors were adjusted with time series structure using MLR with ARMA error. For the six- and 16-year-old groups, all assumptions were met. Thus, the projection of caries-free prevalence until 2030 employed MLR analysis.

### 3.3. Multiple Linear Regression with Arma Errors for 12 Years Old Schoolchildren

Table 3 presents the results of the MLR with ARMA errors. The Ljung Box test shows that the selected model’s residuals are uncorrelated, with a *p*-value = 0.113. Results show that GDP, water fluoridation, and sugar consumption were significantly associated with caries-free prevalence in 12-year-old schoolchildren.

### 3.4. Projection of Caries-Free Prevalence among Schoolchildren

In the projection of caries-free prevalence for six-year-olds, household income, water fluoridation, and sugar consumption were factored in, while GDP, water fluoridation, and sugar consumption were considered for 12- and 16-year-old schoolchildren. Furthermore, the automatic selection model using SPSS version 26 (IBM Corporation, Armonk, New York) Expert Modeler projected the associated factor scores for each year, from 2019–2030, for six-year-olds and from 2020–2030 in the 12- and 16-year-old groups in order to project the prevalence of caries-free until 2030 for each age group. Table 4 shows the projection of caries-free prevalence among 6-, 12- and 16-year-old group until 2030 using multiple linear regression for the 6- and 16-year-old group, and multiple linear regression with ARMA errors for the 12-year-old group.

**Figure 1:**
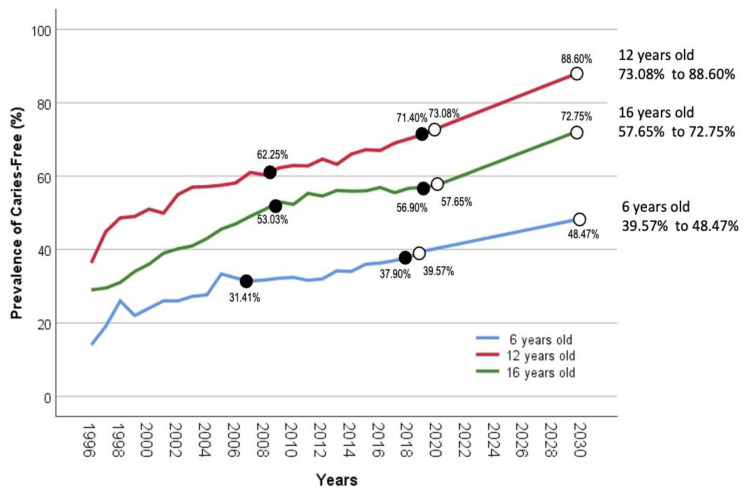
Projection of caries-free prevalence taking into account projected significant associated factors until 2030 among six-, 12-, and 16-year-old schoolchildren in Malaysia.

With the associated factors, the caries-free prevalence was predicted to increase in six-year-old schoolchildren by 9.10% over 11 years, from 39.57% (2019) to 48.47% (2030), as shown in Figure 1. The future increment in the caries-free prevalence among the six-year-old group will slightly improve compared with the past increment of 6.49% over the same duration of 11 years, from 31.41% (2007) to 37.90% (2018). Among 12-year-old schoolchildren, the caries-free prevalence was forecast to increase by 15.52% over ten years, from 73.08% (2020) to 88.60% (2030). This improvement was 1.69 times greater than the past increment of 9.15% over the same period, from 62.25% (2009) to 71.40% (2019). As for 16-year-old schoolchildren, caries-free prevalence would rise by 15.10 % over a decade, from 57.65% (2020) to 72.75% (2030). After considering GDP, sugar consumption, and water fluoridation, the projected prevalence of caries-free will increase by almost four times more than a 3.87% improvement over the same period, from 53.03% (2009) to 56.90% (2019).

## 4. Discussion

In this study, caries-free prevalence is projected to increase in all age groups of schoolchildren, with a higher increment of caries-free prevalence observed in the next decade until 2030 compared with the past decades in all age groups. The economic factors, i.e., household income and GDP, sugar consumption, and water fluoridation, were significantly associated with caries-free prevalence, with a highly positive correlation in all age groups.

After considering the effects of GDP, sugar consumption, and water fluoridation, the highest increment observed in the projected caries-free prevalence was 15% among the 12- and 16-year-old schoolchildren. Furthermore, the caries-free prevalence over the next decade was predicted to be consistently the highest among the 12-, followed by the 16-year-old schoolchildren, while the lowest was among the six-year-olds. However, while the numbers of 12- and 16-year-old schoolchildren have steadily increased and achieved the national targets of 70% and 50% caries-free in 2020, the caries-free prevalence in six-year-old children has yet to achieve the national target of 50% caries-free among this age group by 2020, even in 2030.

The low projected prevalence of caries-free in the six-year-old group could be correlated with a highly sugary diet. According to the local studies by Ruhaya, et al. [40] and Amarra, Khor and Chan [11], schoolchildren in Malaysia consume a high-sugar diet, with sweetened foods for snacking and refreshment. The preschoolers in Malaysia consumed sweet foods and beverages more than two times a day, which exceeded the recommendation by the WHO [10]. Therefore, the high-sugar diet has influenced the reduction of caries-free prevalence among the population, as discussed in many studies [41,42,43,44]. Thus, unless there are vigorous and effective public health initiatives to control sugar consumption among younger children, the projection of caries-free children in this vulnerable age group will remain low until 2030.

Additionally, poor utilisation of primary care services among preschoolers may account for the low prevalence of caries-free in six-year-old schoolchildren. One of the reasons is that the services provided need to be more evenly distributed across Malaysia [45]. As a result of poor service utilisation, a highly sugary diet, and poor oral health behaviours, patients may have developed caries when they start primary school, where the school dental services start. Unfortunately, during this time, caries might progress to an advanced stage where the damage is irreversible and eventually might lead to tooth loss [15,46].

On the other note, many factors have contributed to the high caries-free prevalence projected for 12-year-old schoolchildren in Malaysia. Apart from natural causes, such as newly erupted and less exposed permanent teeth [47], along with the positive impacts of water fluoridation and economic factor, the achievement is mainly attributed to the established IDC by the School Dental Services (SDS) team for primary school children [48]. SDS covers everything from preventive to curative treatment rendered to this age group to ensure they are orally fit [49].

In the 16-year-old group, the four times greater prevalence improvement than in the past ten years from 2009 to 2019 may reflect the positive impacts of oral health promotion programmes and new approaches targeted to adolescents in recent years by the government. For example, various social media platforms, i.e., Facebook and Instagram, are intensively used, with more interactive oral health activities conducted with a smaller group of adolescents in recent years [50].

Nevertheless, the national oral health plan to achieve 50% caries-free in six-year-olds still needs more work, even after the projected caries-free prevalence in 2030. It highlights the opportunity to revise or strengthen the oral health programmes targeted for this group, for example, by improving the programmes for preschoolers, which will directly impact the oral health of six-year-old schoolchildren. More holistic and integrated strategies with more resources prioritised for this age group could be explored. In addition, the active involvement of critical stakeholders such as teachers and parents can be reinforced. For example, a new intervention programme in Perak, Malaysia, the Beautiful Smile for All Programme (SIMSP), introduced in 2019, encourages other stakeholders’ active involvement, i.e., dental therapists, teachers and parents. As a result, SIMSP has successfully lowered dental plaque scores, reduced carbonate drinks, and improved parents’ oral health knowledge and health literacy compared with the preschool oral healthcare programme involving only a dental therapist. The findings proved the positive effects of stakeholders’ engagement in young children’s oral health [51]. As the effect of caries is cumulative, successful prevention strategies in this age group will indirectly lead to better caries prevalence in older age groups as well [52].

The economic factors, i.e., household income and GDP, were associated with caries-free prevalence among six-year-olds, 12-, and 16-year-olds, respectively. This finding concurs with studies that reported that the country’s economic performance affected oral health [24]. A meta-analysis study that confirmed household income is associated with caries-free [53] suggested that underprivileged individuals with socio-economic difficulties usually experience more caries. This is because most of them are uninsured and unable to afford the cost of the intervention, which restricts them from regular dental care, leaving them untreated and leading to colossal health disparity among the high- and low-income populations [54]. Moreover, their economic status has affected their diet and oral health practises, such as tooth brushing behaviour, reflecting health disparities between different social classes [55].

Simultaneously, studies also found that GDP growth is also related to increased health expenditure, better healthcare facilities, and a boost in human capital productivity, ultimately contributing to more economic growth [56,57]. For example, in China, the consistent increasing projection trend of caries-free children from 44.2% (2014) to 48.8% (2018) was associated with economic growth, where the GDP per capita increased from 1112 Renminbi (RMB) in 1987 to 38,420 RMB in 2012. The rise in GDP per capita resulted in more significant government investment in public health, leading to a larger workforce, better health services, and greater accessibility nationwide, hence improving caries-free prevalence [58].

Meanwhile, water fluoridation has been established as the most popular and cost-effective preventive strategy, disseminating the benefits to a large population regardless of social status [59,60]. Due to widespread water fluoridation coverage in Malaysia, most children were consistently exposed to fluoride during tooth formation, which might explain the high prevalence of caries-free among 12-year-old schoolchildren compared with older children whose teeth had been exposed to an oral environment for a longer time. This also explains the high prevalence of caries-free among 12-year-olds in Malaysia compared with the same age children in neighbouring countries, such as Indonesia [61] and Thailand [62], with no water fluoridation programme. Thus, the coverage should be expanded, especially in remote areas with limited access to oral care. Even though sugar is the most crucial determinant of caries, it was only significant among the 16 year-old-group in multivariable analysis but not in correlation analysis. Unlike in the younger groups, sugar consumption was significant in both analyses. This finding concurs with a meta-analysis study on dietary intake, which concluded a substantial reduction in added sugar consumption during the transition age from adolescence into early adulthood. The changes in taste preference with less sucrose concentrations in foods than in adolescence suggested a universal physiological mechanism where preference for sugary foods decreases with age [63].

In comparison, literature worldwide suggests that during adolescence, an individual starts to stay outdoors for a more extended period without parents’ supervision and begins to develop self-made decisions regarding diet preference and oral hygiene [64,65]. Therefore, their immature decision-making and poor oral health awareness could drastically reduce caries-free prevalence in 16-year-olds compared with 12-year-olds. Furthermore, evidence suggested a drastic progression of caries incidence from 11 to 16 years due to more freedom in oral health behaviour. Its progression becomes severe from age 15 to 20, where half of the initial proximal lesion progresses to a cavity [63].

However, due to the unavailability of periodical data, oral health behaviours and accessibility could not be analysed. The challenges should not hinder us from conducting the first projection study of caries-free prevalence and the associated factors among Malaysian schoolchildren. Within the limitation, we could provide the baseline projection data. This study highlights the need for periodical data collection on oral disease determinants. One main strength of this study is the generalisability of the findings, as the sample was nationwide data involving all age groups of Malaysian schoolchildren. This study included all the schoolchildren attending government-registered primary and secondary schools involved in Incremental Dental Care for school dental services rendered by the Ministry of Health. Hence, the sample represents the schoolchildren population in Malaysia. Furthermore, the sample size estimation was not applicable as the time series analysis needed the data points to be as large as possible to estimate a model accurately, and this study employed a total sampling method and sampled all available data points on caries-free prevalence for each age group.

## 5. Conclusions

The caries-free prevalence in Malaysian schoolchildren over the next decade is projected to increase and remain the highest in 12-year-olds, followed by 16- and six-year-old groups, with the increment multiplied the highest among the 16-year-old schoolchildren. Economic factors, sugar consumption, and water fluoridation significantly contribute to future caries-free prevalence, with the most contributing factor being water fluoridation. Thus, strategic oral health plans to recognise and maintain effective promotion programmes and strengthen others for each age group are crucial towards attaining the goals of the FDI World Dental Vision to deliver optimal and comprehensive oral healthcare for all by 2030.

## Figures and Tables

**Table 1:** Correlation test between potential associated factors and caries-free prevalence.

Age Group	Correlation Pair	Correlation Coefficient (r)	*p*-Value *
6 years old	GDP vs. caries-free	0.841	<0.001
	CPI vs. caries-free	0.901	<0.001
	Household income vs. caries-free	0.819	<0.001
	Relative poverty vs. caries-free	−0.693	<0.001
	Sugar consumption vs. caries-free	−0.505	0.014
	Fluoridated water vs. caries-free	0.790	<0.001
12 years old	GDP vs. caries-free	0.893	<0.001
	CPI vs. caries-free	0.943	<0.001
	Household income vs. caries-free	0.869	<0.001
	Relative poverty vs. caries-free	−0.698	<0.001
	Sugar consumption vs. caries-free	−0.391	0.050
	Fluoridated water vs. caries-free	0.771	0.001
16 years old	GDP vs. caries-free	0.881	<0.001
	CPI vs. caries-free	0.936	<0.001
	Household income vs. caries-free	0.840	<0.001
	Relative Poverty vs. caries-free	−0.686	<0.001
	Sugar consumption vs. caries-free	−0.340	0.104
	Fluoridated water vs. caries-free	0.867	<0.001

Note: Gross domestic product (GDP); Consumer price index (CPI); * Level of significance = 0.050.

**Table 2:** Results of MLR of caries-free prevalence and its associated factors.

Age	Associated Factors	Coefficient (95% CI)	Standard Error	*p*-Value *	VIF	R^2^
6	Household Income	2.6 × 10^−3^ (0.002, 0.003)	3.6 × 10^−4^	<0.001	2.13	0.945
	Sugar consumption	−0.06 (−0.08, −0.05)	0.007	<0.001	1.03	
	Water fluoridation	0.34 (0.16, 0.53)	0.089	0.001	2.17	
12	GDP	1 × 10^−3^ (8 × 10^−4^, 1.4 × 10^−3^)	1.3 × 10^−4^	<0.001	2.16	0.931
	Sugar consumption	−0.07 (−0.09, −0.04)	0.012	<0.001	1.02	
	Water fluoridation	0.31 (0.01, 0.61)	0.144	0.044	2.20	
16	GDP	9.0 × 10^−4^ (0.001, 0.002)	1.2 × 10^−4^	<0.001	2.16	0.949
	Sugar consumption	−0.06 (−0.08, −0.03)	0.011	<0.001	1.02	
	Water fluoridation	0.78 (0.49, 1.07)	0.138	<0.001	2.20	

Note: Gross domestic product (GDP); * Level of significance = 0.050.

**Table 3:** Results of MLR with ARMA errors of caries-free prevalence and its associated factors.

Model	Age Group	Variables	Coefficients	SE	*p*-Value	Ljung Box Test (*p*-Value)
Regression with ARMA (0,0,1) Errors	12 years old	GDP Water fluoridation	0.0012 0.215	0.0002 0.177	<0.001 <0.044	0.113
		Sugar & sweetener	−0.063	0.013	0.044	

Note: Gross domestic product (GDP).

**Table 4:** Projected prevalence of caries-free up to 2030 among 6, 12 and 16-years old school children.

Age Group	Forecasted Years	Significant Associated Factors			Prevalence of Caries-Free (%)
		Household Income (MYR) (Brown Linear)	Sugar Consumption (kcal/Capita/Day) (Damped Trend)	Water Fluoridation (%) (ARIMA 0,1,0)	
6 years old	2019	5873.00	413.59	74.62	39.57
	2020	6088.00	414.02	75.15	40.37
	2021	6303.00	414.39	75.67	41.17
	2022	6518.00	414.70	76.19	41.97
	2023	6733.00	414.98	76.71	42.78
	2024	6948.00	415.21	77.24	43.59
	2025	7163.00	415.42	77.76	44.40
	2026	7378.00	415.59	78.28	45.21
	2027	7593.00	415.74	78.80	46.02
	2028	7808.00	415.87	79.33	46.84
	2029	8023.00	415.98	79.85	47.66
	2030	8238.00	416.08	80.37	48.47
		GDP (Million MYR) (Brown Linear)	Sugar consumption (kcal/capita/day) (Damped trend)	Water Fluoridation (%) (Damped trend)	
12 years old	2020	40,439.16	413.37	73.35	73.08
	2021	41,685.26	413.82	73.80	74.69
	2022	42,931.36	414.20	74.24	76.22
	2023	44,177.45	414.53	74.69	77.76
	2024	45,423.55	414.82	75.14	79.30
	2025	46,669.65	415.06	75.59	80.85
	2026	47,915.75	415.27	76.03	82.39
	2027	49,161.85	415.45	76.48	83.94
	2028	50,407.95	415.61	76.93	85.49
	2029	51,654.05	415.75	77.38	87.04
	2030	52,900.15	415.86	77.83	88.60
		GDP (Million MYR) (Brown Linear)	Sugar consumption (kcal/capita/day) (Damped trend)	Water Fluoridation (%) (Damped trend)	
16 years old	2020	40,439.16	57.65	73.35	57.65
	2021	41,685.26	59.15	73.80	59.15
	2022	42,931.36	60.65	74.24	60.65
	2023	44,177.45	62.16	74.69	62.16
	2024	45,423.55	63.66	75.14	63.66
	2025	46,669.65	65.18	75.59	65.18
	2026	47,915.75	66.68	76.03	66.68
	2027	49,161.85	68.20	76.48	68.20
	2028	50,407.95	69.71	76.93	69.71
	2029	51,654.05	71.23	77.38	71.23
	2030	52,900.15	72.75	77.83	72.75

Note: Gross domestic product (GDP).

## Data Availability

Not applicable.

## Supplementary Materials

The following supporting information can be downloaded at: https://www.mdpi.com/article/10.3390/children10071125/s1, Table S1: Test of assumptions on model.
