# Supplementary material for: Multivariable Projections of Caries-Free Prevalence and the Associated Factors from 2019 to 2030 among Schoolchildren Aged 6, 12 and 16-Year-Old in Malaysia
Source: Children (Basel). 2023 Jun 28;10(7):1125. doi: 10.3390/children10071125 (PMC10378140; doi:10.3390/children10071125)
Supplement: Supplementary file 1 [file children-10-01125-s001.zip › children-2277601-supplementary.pdf]

Table S1. Test of assumptions on model.

Age group	Assumption	*p*-value	Summary
6 years old	Normality (Kolmogrov-Smirnov)	0.200	The *p*-values were not significant. There was no violation of assumptions of normality, linearity, serial correlation and equal variance.
	Linearity (Runs test)	1.000	
	Serial Correlation (Breusch-Godfrey)	0.270	
	Homosdesticity (Breusch-Pagan)	0.293	
12 years old	Normality (Kolmogrov-Smirnov)	0.200	The *p*-values in serial correlation was significant. The null hypothesis was rejected. The errors were serially correlated. The other assumptions of normality, linearity, and equal variance were met.
	Linearity (Runs test)	0.861	
	Serial Correlation (Breusch-Godfrey)	0.049	
	Homosdesticity (Breusch-Pagan)	0.293	
16 years old	Normality (Kolmogrov-Smirnov)	0.200	The *p*-values were not significant. There was no violation of assumptions of normality, linearity, serial correlation and equal variance.
	Linearity (Runs test)	0.060	
	Serial Correlation (Breusch-Godfrey)	0.176	
	Homosdesticity (Breusch-Pagan)	0.138	
